# Impact of Dorn Therapy on a Patient With a Prolapsed Intervertebral Disc: A Case Report

**DOI:** 10.7759/cureus.29023

**Published:** 2022-09-11

**Authors:** Saloni Shah, Samiksha Khemani, Shrikant Mhase, Sabih N Khan, Aishwarya A Pashine, Manoj S Patil

**Affiliations:** 1 Rehabilitation, Mahatma Gandhi Mission (MGM) School of Physiotherapy, Aurangabad, IND; 2 Department of Community Physiotherapy, Mahatma Gandhi Mission (MGM) School of Physiotherapy, Aurangabad, IND; 3 Department of Cardiorespiratory Physiotherapy, Mahatma Gandhi Mission (MGM) School of Physiotherapy, Aurangabad, IND; 4 Research, NKP Salve Institute of Medical Sciences and Research Centre, Nagpur, IND; 5 Research and Development, Jawaharlal Nehru Medical College, Datta Meghe Institute of Medical Sciences, Wardha, IND

**Keywords:** case report, dorn therapy, prolapsed intervertebral disc, nucleus pulposus herniation, disc bulge

## Abstract

A prolapsed intervertebral disc (PIVD) refers to herniation of the nucleus pulposus out of the intervertebral space. Mechanical nerve compression brought on by a protruding nucleus pulposus and an increase in chemokines in the affected area causes the underlying pain, which is typically treated conventionally with surgery or medication. Nonetheless, these management strategies may not always be effective and may impact a person’s quality of life. This report highlights the case of a 31-year-old male patient, who complained of neck and low back pain associated with tingling sensations for two months, and was treated with Dorn Therapy (DT) which is the basis of pain management intervention in conjunction with spinal traction, spinal mobilization, stretching, muscular strength training, and transcutaneous electrical nerve stimulation. Following a week of pain management techniques, DT significantly reduced pain, improved range of motion, and enhanced muscle strength. This case report highlights the impact of DT on pain associated with a PIVD, which may curtail an individual’s well-being.

## Introduction

Herniated cervical and lumbar discs are the most frequent causes of neck and back discomfort as the intervertebral disc permits a wide range of movement in all directions and acts as a shock-absorbing cushion for the axial loads of the body [1,2]. The spine serves the body in a number of important ways, including providing structural support, protecting the spinal cord and branching spinal neurons, and facilitating flexibility and mobility [2]. The incidence of a herniated disc is about 5 to 20 cases per 1000 adults annually and is most common in people in their third to fifth decade of life, with a male-to-female ratio of 2:1 [3].

Disc herniation is thought to be caused by a number of changes in the biology of the intervertebral disc, including water retention in the nucleus pulposus (NP), an increase in the percentage of type I collagen in the NP and inner annulus fibrosus, the breakdown of collagen and extracellular matrix components, and the upregulation of degradation pathways such as apoptosis, matrix metalloproteinase expression, and inflammatory pathways [4].

Dorn Therapy (DT) is an efficacious, safe, and non-manipulative therapy that originated in Germany by Dieter Dorn in 1973. This manual therapy intends to carefully correct misalignments in vertebrae and joints [5]. Spinal misalignments may cause nerve compression which can cause various disorders. The method relaxes the muscles of the back so that the skeletal structure is able to reposition and balance itself. It involves the application of gentle pressure and correction in dynamic motion [6]. Hence, this novel case report emphasizes the execution of DT for the pain associated with a prolapsed intervertebral disc (PIVD) and appraises its impact.

## Case presentation

Patient and observation

A 31-year-old male, presented with the main complaints of neck and low back pain which was gradual in onset, continuous in nature, and aggravated with activity over four years. He first felt pain with a tingling sensation in the cervical region at C5-C6, but after two years, he started complaining of pain in the lumbar region at L5-S1. The left side of the cervical and lumbar region was more painful so he consulted an orthopaedic doctor who recommended he take physiotherapy treatment. On observation, marked postural deviation with a forward-head posture was seen.

Clinical findings

On physical examination, all his vital parameters including temperature (98.6°F), pulse rate (72 beats per minute), SPO_2_ (97%), and blood pressure (140/90 mmHg) were within the normal limits. The pain intensity recorded on the numerical pain rating scale (NPRS) was 7 on activity and 4 at rest. Further, on palpation, the C-5 region exhibited grade 1 tenderness along with the presence of a trigger point just above the superior angle of the left scapula, as well as tightness of the upper trapezoid, hamstring, and piriformis muscles. Additionally, on assessment, range of motion (ROM) for cervical extension, side rotation, and lumbar extension of the left side was limited and painful. The muscle strength was grade 3+ for cervical and lumbar flexors whereas, grade 3 for cervical and lumbar extensors and side rotators of the left side.

Diagnostic assessment

**Figure 1:**
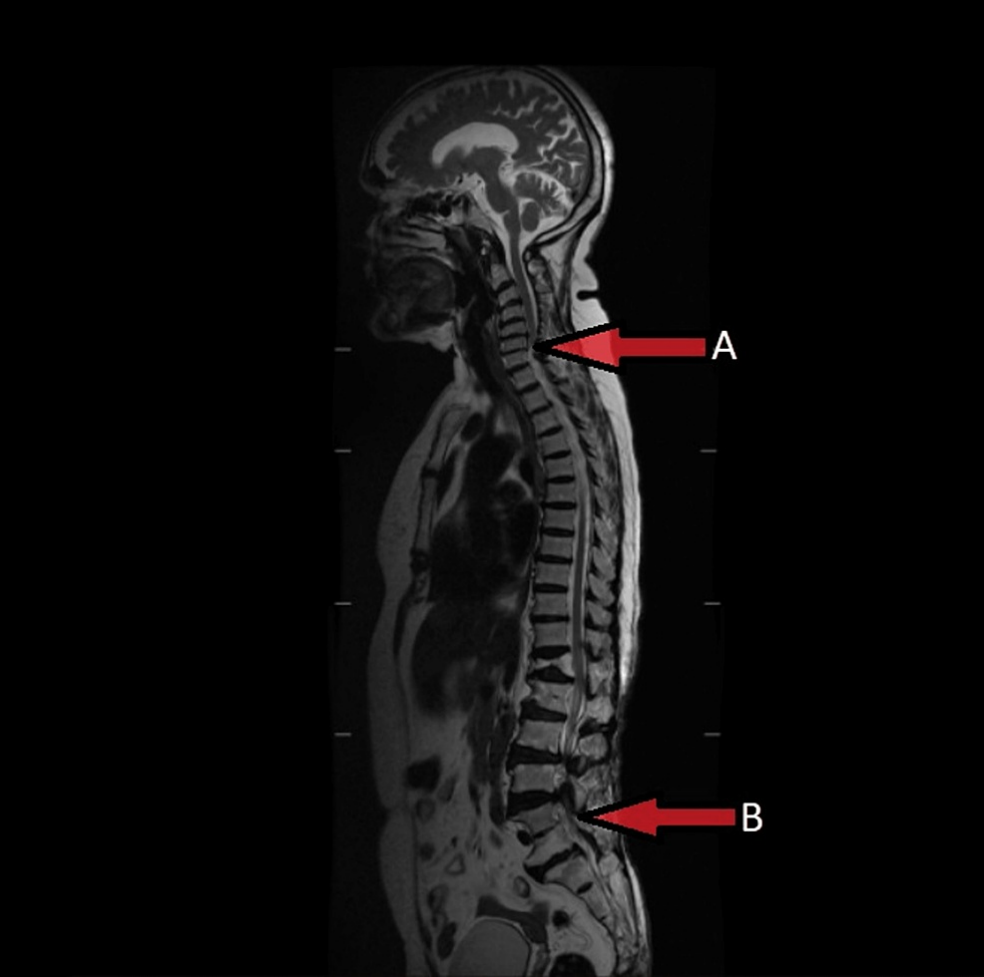
Magnetic Resonance Imaging (MRI) of the spine. A= Desiccation and mild disc bulge at C5-C6 region, B= Intruding thecal sac at L5-S1 region.

The patient underwent Magnetic Resonance Imaging (MRI) for screening of the spine that revealed desiccation and a mild bulge of the C5-C6 and L5-S1 disc intruding on the thecal sac (Figure 1).

Therapeutic interventions

Before commencing physiotherapeutic intervention (PI), the patient was briefed about the intervention protocol, and written informed consent was received. The patient was made aware that his medical records will be kept confidential. The PI mainly focussed on DT along with cervical and lumbar traction which was given with the traction machine which helps in managing pain by decompression to relieve pressure on the spine and was performed everyday for 20 minutes [7], followed by stretching which was performed 3 times per day, involving elongation time of 10 seconds with rest intervals of 3 seconds between each elongation, and total number of 3 elongations were performed which helps in improving flexibility, piriformis muscle energy techniques, a manual treatment method that relaxes and lengthens muscles by using the body's own energy in the form of moderate isometric contractions, which serves to improve musculoskeletal function [8], spinal mobilization to enhance joint function, transcutaneous electrical nerve stimulation which reduces symptomatic pain by stimulating pain gate mechanism and William’s flexion exercises which include pelvic tilt, hamstring stretch, hip flexor stretches, single knee-to-chest, double knee-to-chest, partial sit-ups and squats, each session took about one hour in which duration of each exercise was 8 to 10 seconds for each set with each set consisting of 10 repetitions and with total number of 3 sets performed each day to improve lumbar flexion and strengthen gluteus and abdominal muscles along with strengthening exercises was continued for one week [9,10].

**Figure 2:**
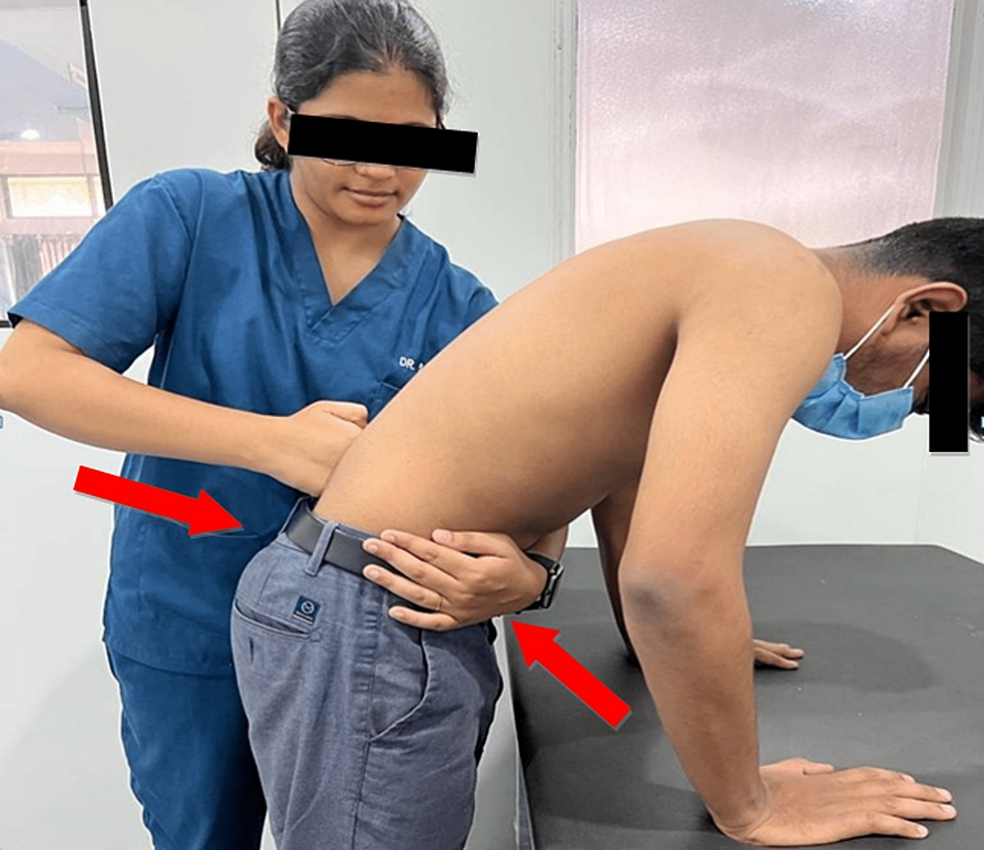
Demonstration of Dorn Therapy.

The therapist performed the DT by standing lateral to the patient and stabilizing the patient's pelvis with the left hand and with the knuckles of the right hand applying slight pressure to the spinous process of the lower lumbar vertebrae while maintaining the pressure simultaneous lumbar flexion and extension is performed (Figure 2). Three sets of ten repetitions each were performed with interval periods of relaxation and assessment [6].

Follow-up and outcome of interventions

During the initiation of treatment, pain intensity reported was 7 on NPRS, whereas on termination, it was reported as 1. The patient overall completed 7 days session after which ROM and muscle strength of the cervical and lumbar spine [11,12] showed appreciable increments, as shown in Table 1.

**Table 1:** Outcomes describing pre-treatment and post-treatment values.

OUTCOMES	Pre-treatment values	Post-treatment values
Pain	7 on the numerical pain rating scale	1 on the numerical pain rating scale
Range of motion (cervical region) in degrees using a goniometer		
Flexion	0-34	0-40
Extension	34-0	40-0
Left lateral flexion	0-54	0-54
Left cervical rotation	0-65	0-70
Range of motion (lumbar region) in centimeters (cms) using modified-modified Schober’s test		
Flexion	2 cm	3 cm
Extension	1 cm	2 cm
Left thoracolumbar rotation in degrees using a goniometer	0-20	0-25
Muscle strength for cervical and lumbar regions in grades by manual muscle testing		
Flexors	3+	4
Extensors	3	4
Left and right side rotators	3	4

## Discussion

The uniqueness of this case report demonstrates the application of DT and its positive impact on PIVD patients with chronic low back pain (LBP). DT aims to make patients functionally independent and have a better quality of life. Practitioners of DT consider the movement a crucial component of treatment because all the corrections occur dynamically. Movement stretches the muscles so that they are unable to resist the correction, thus releasing tension and maintaining muscle length. Muscle flexibility is provided through the mechanical principles of counter pressure and leverage forces with the body usually accepting this type of correction easily in conjunction with the patient's active involvement. Murugan et al. illustrated that DT followed by other PI proved beneficial and provided immediate relief of chronic LBP caused by lumbar spondylosis [6], which correlates well with this case report as DT significantly reduced pain intensity and enhanced ROM as well as muscle strength.

Along with DT, other interventions mainly involved traction and William’s flexion exercises. A recent meta-analysis suggested that in individuals with herniated discs, mechanical traction can effectively alleviate lumbar and leg pain, but has no noticeable impact on spinal motion. Additionally, it reported that mechanical traction has a therapeutic impact that is noticeably superior to that of traditional physical therapy [13], whereas William's flexion exercises involving a series of components are also effective in reducing pain and disability as given in a narrative review by Kim et al. [9]. Both interventions combined with DT boosted the intervention protocol described in this case report.

DT with almost no risk requires a very short treatment period in a session. The reported outcomes suggest that DT can be given to patients with chronic LBP unless noted with any serious illness. However, this therapy in correlation with home exercises and ergonomic advice enhances patients’ confidence, reduces the risk of injury, and improves the quality of life [6].

## Conclusions

This case report outlines the beneficial impact of DT in chronic low back pain PIVD patients as there is a substantial reduction in pain intensity along with appreciable improvement in ROM and muscle strength. However, it can be suggested to use it in combination with conventional PI for the immediate relief of chronic LBP. Since this is a case report, it is impossible to extrapolate the findings to a larger sample size. As a result, further research is required to investigate the use of DT and its effectiveness to better understand the treatment parameters.
